# Inter-study reproducibility of circumferential strain and strain rates at 1.5T and 3T: a comparison of tagging and feature tracking

**DOI:** 10.1186/1532-429X-16-S1-P354

**Published:** 2014-01-16

**Authors:** Anvesha Singh, Christopher D Steadman, Jamal N Khan, Sheraz A Nazir, Prathap Kanagala, Gerry P McCann

**Affiliations:** 1 Cardiovascular Sciences, University of Leicester, Leicester, Leicestershire, UK; 2 Cardiology, Glenfield Hospital, Leicester, Leicestershire, UK; 3 Cardiology, Poole Hospital NHS Foundation Trust, Poole, UK

## Background

Feature Tracking (FT) is a relatively new technique for measuring strain on cardiac magnetic resonance imaging (CMR), that has been shown to have reasonable inter-study reproducibility (Coefficient of variation (CoV) ~20%) in healthy volunteers. The inter-study reproducibility of FT has not yet been reported in any patient groups, nor compared to that of MRI tagging. We sought to determine the inter-study reproducibility of circumferential strain and strain rates using FT and tagging at 1.5T and 3T scanners, in patients with moderate-severe Aortic Stenosis (AS).

## Methods

CMR was performed twice in 8 patients with severe AS on a 1.5T scanner and 10 patients with moderate-severe AS at 3T. Three short-axis tagged images were acquired, in addition to the standard SSFP short-axis cine stack. InTag (Creatis, Lyon, France) in OsiriX (Geneva, Switzerland) was used to calculate the Circumferential Peak Systolic Strain (PSS), Peak Systolic Strain Rate (PSSR) and Peak Early Diastolic Strain Rate (PEDSR). Diogenes CMR FT (TomTec Imaging Systems, Munich, Germany) was used to calculate the same parameters on nearest SSFP cine images.

## Results

**Figure 1:**
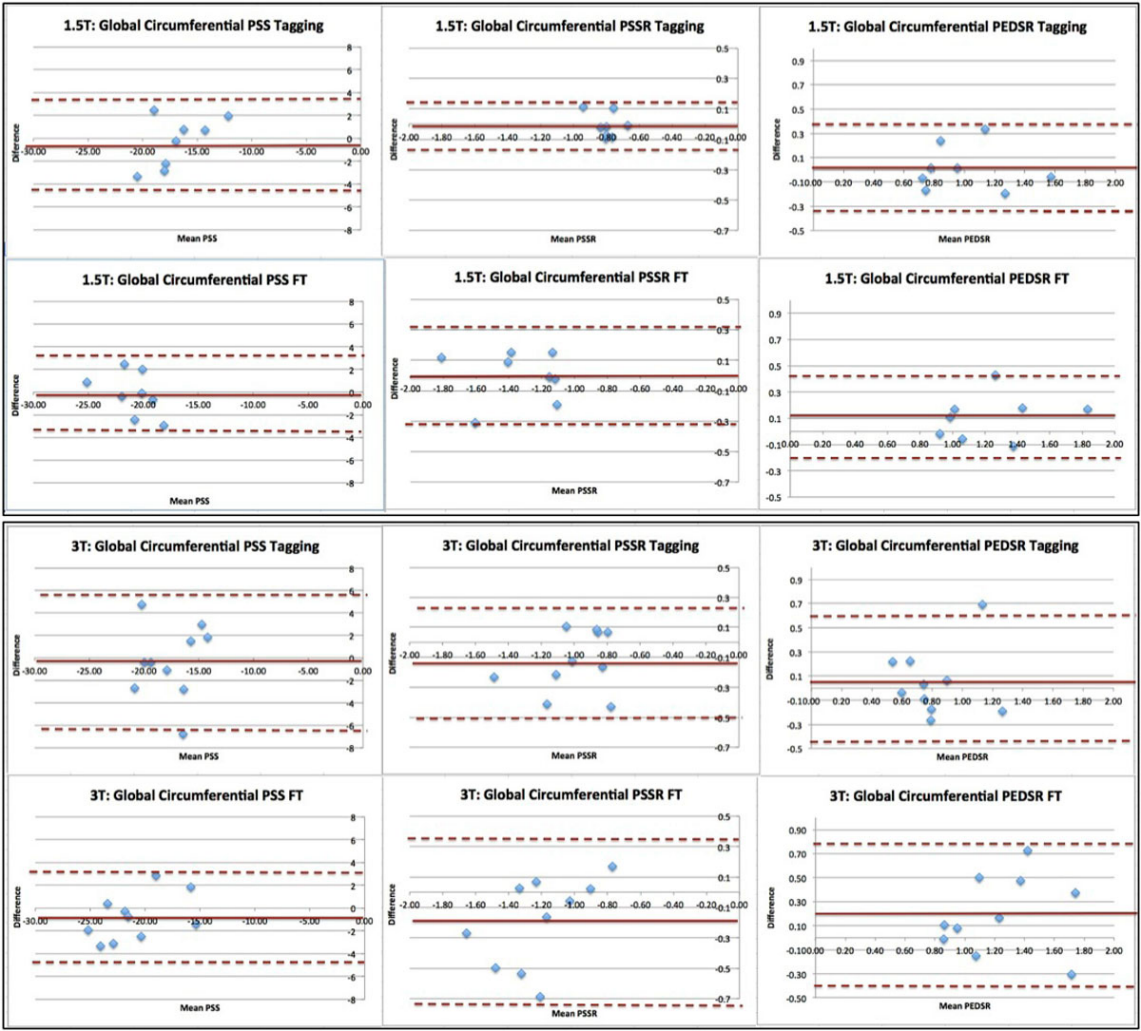


Overall, FT gave higher strain and strain rate values when compared to tagging. On paired sample t-tests, there was no significant difference in the strain and strain rate values between scan one and scan two, using both tagging and FT, at both 1.5T and 3T. The inter-study reproducibility of both techniques was higher at 1.5T compared to 3T. (Table 1, Figure 1) Comparing tagging vs FT, PSS was more reproducible with FT at both 1.5T and 3T, while PSSR was more reproducible with tagging. PEDSR demonstrated similar inter-study reproducibility using both techniques, but was much more reproducible at 1.5T than 3T. (CoV's for circumferential PSS, PSSR and PEDSR at 1.5T- FT: 8.6, 11.8 and 13.1%, tagging: 12.2, 9.4 and 17.5%; CoV's at 3T-FT: 9.4, 23 and 25.6%, tagging: 17.9, 19.3 and 32.5%).

**Table 1:** Inter-study reproducibility of global Circumferential strain and strain rates using Tagging and FT on 1.5T and 3T scanners.

Scanner	Technique	Parameter	Average Value+	Paired Mean Difference (SD)	Limits of agreement	R (Pearson's Correlation)	CoV
**1.5T**	Tagging	PSS	-16.86 ± 2.78	-0.33 (2.06)	-4.36 to 3.70	0.78*	12.2

		PSSR	-0.80 ± 0.08	-0.01 (0.07)	-0.16 to 0.14	0.60	9.4

		PEDSR	1.00 ± 0.31	0.02 (0.18)	-0.33 to 0.36	0.82*	17.5

	FT	PSS	-20.88 ± 2.26	-0.14 (1.81)	-3.68 to 3.39	0.70	8.6

		PSSR	-1.34 ± 0.27	0.00 (0.16)	-0.31 to 0.31	0.81*	11.8

		PEDSR	1.24 ± 0.31	0.11 (0.16)	-0.21 to 0.43	0.86*	13.1

**3T**	Tagging	PSS	-17.59 ± 2.86	-0.30 (3.14)	-6.46 to 5.86	0.36	17.9

		PSSR	-0.99 ± 0.25	-0.13 (0.19)	-0.50 to 0.25	0.67*	19.3

		PEDSR	0.82 ± 0.26	0.05 (0.27)	-0.47 to 0.57	0.46	32.5

	FT	PSS	-20.94 ± 3.43	-0.82 (1.97)	-4.67 to 3.04	0.86*	9.4

		PSSR	-1.21 ± 0.31	-0.19 (0.28)	-0.74 to 0.35	0.59	23.0

		PEDSR	1.23 ± 0.37	0.20 (0.30)	-0.40 to 0.79	0.61	25.6

Abbreviations: PSS: peak systolic strain, PSSR: peak systolic strain rate, PEDSR: peak early diastolic strain rate, CoV: Coefficient of variation. Average of epicardial and endocardial contours used for FT. No significant difference between scan-1 and scan-2 values on paired sample t-test; *Statistically significant correlation (p < 0.05), + Average of all values from scan 1 and 2.

## Conclusions

Both tagging and FT have good reproducibility at 1.5T and modest reproducibility at 3T scanners. This may partly be due to greater artefacts at 3T. Overall, FT appears to have higher reproducibility than tagging for circumferential PSS, while PSSR is more reproducible with tagging. If the main parameter of interest is PEDSR, scanning at 1.5T and using FT is more preferable. Given that FT does not require additional image acquisitions and involves shorter post-processing time, this technique is likely to become the preferred method for strain and strain rate quantification with CMR.

## Funding

This work is part of a Project Grant funded by the British Heart Foundation (PG/07/068/2334). Support also received from the NIHR Leicester Cardiovascular Biomedical Research Unit.

